# Trauma, firearms, and pregnancy: Beyond the cushion^☆^

**DOI:** 10.1016/j.sopen.2025.04.003

**Published:** 2025-04-17

**Authors:** Flora S. Park, Jeffry Nahmias, Jordan Shin, Ji Young Li, Cristobal Barrios, Sebastian Schubl, Areg Grigorian

**Affiliations:** Department of Surgery, Division of Trauma, Burns and Surgical Critical Care, University of California, Irvine School of Medicine, 3800 Chapman Ave. #6200, Orange, CA 92868, USA

**Keywords:** Penetrating trauma, Pregnancy, Outcomes, Gastrointestinal injury

## Abstract

**Background:**

Pregnancy introduces unique anatomical and physiological changes that could influence injury susceptibility, including redistribution of abdominal organs and adipose tissue that could provide a protective “cushion effect” against penetrating trauma. This study aimed to understand how injury patterns and clinical outcomes differ between pregnant trauma patients (PTPs) and their non-pregnant counterparts presenting after penetrating trauma, hypothesizing that PTPs have a lower mortality risk and incur less severe abdominal injuries due to the proposed cushion effect.

**Methods:**

The 2020–2021 Trauma Quality Improvement Program database was queried for all female patients <50-years-old presenting after penetrating trauma. The primary outcome was mortality, and secondary outcomes included the incidence of gunshot wounds and abdominal injuries. We compared pregnant versus non-pregnant patients with bivariate and multivariable logistic regression analyses.

**Results:**

From 25,812 female patients, 531 (2.1 %) were PTPs. Gunshot wounds were more prevalent among PTPs (64.2 % vs. 46.0 %, *p* < 0.001), and PTPs exhibited a higher incidence of gastrointestinal injuries (8.5 % vs. 6.3 %, *p* = 0.04). PTPs also had more in-hospital cardiac arrests (2.1 % vs. 1.0 %, *p* = 0.02) and increased mortality (7.5 % vs. 5.6 %, *p* = 0.052) however similar associated risk of death (OR = 1.08, CI 0.65–1.77, *p* = 0.78) compared to non-pregnant patients.

**Discussion:**

The prevalence of firearm violence emphasizes the need for targeted primary preventive strategies for PTPs. Furthermore, the heightened vulnerability of PTPs to gastrointestinal injuries following penetrating trauma, potentially attributed to the gravid uterus's spatial changes, challenges any proposed “cushion effect.”

## Highlights

• Increased gastrointestinal injury rate in pregnant penetrating trauma patients
• Similar mortality rate in pregnant and non-pregnant penetrating trauma patients
• Higher incidence of gunshot wounds in pregnant patients
• Necessity for increased firearm and substance use counseling in pregnancy

## Introduction

Trauma stands as a predominant non-obstetric cause of maternal mortality in the United States, representing >40 % of maternal deaths [[1], [2], [3]]. Pregnant trauma patients (PTPs) experience a mortality rate nearly twice as high as their non-pregnant counterparts [4]. Notably, penetrating injuries, such as gunshot wounds (GSWs) and stab wounds, account for approximately 2–7 % of these traumatic incidents [5].

The unique physiological and anatomical changes that occur during pregnancy can alter a woman's vulnerability to trauma. It is suggested that pregnant patients face an elevated risk for abdominal trauma, a risk that escalates as the pregnancy progresses. This may be due to the growth of the gravid uterus and the anatomic displacement of intraperitoneal organs which is thought to increase the likelihood of harm to both the mother and fetus [2,3]. In contrast, there exists a theory suggesting that the amniotic fluid provides a protective cushion for the fetus and uterus, particularly between 13 and 23 weeks of gestation [6]. Moreover, the gestational redistribution of body fat, particularly around the upper abdomen, might offer a protective barrier, though this hypothesis has seen varying levels of support [6,7]. Additionally, some have hypothesized that mortality in PTPs is less likely due to the displacement of abdominal viscera [7]. However, these theories have only been suggested in the context of blunt PTPs, and they have not been explored in penetrating PTPs. The varied perspectives on injury outcomes in PTPs with penetrating trauma highlights the need for additional investigation into this specific at-risk patient population.

The objective of this study is to examine injury patterns and clinical outcomes in PTPs with penetrating trauma. Based on the previously posited “cushion effect” with blunt trauma, we hypothesize that PTPs with penetrating trauma also face a decreased risk of mortality and have less severe abdominal injuries compared to non-pregnant patients due to the redistribution of abdominal organs and adipose tissue.

## Materials and methods

In this retrospective cohort study, the American College of Surgeons Trauma Quality Improvement Program (ACS-TQIP) database was queried (2020-2021) for all female patients <50-years-old presenting after penetrating trauma. This study was granted a waiver of consent by our local Institutional Review Board as the study utilizes a deidentified national database. We excluded all male patients, blunt mechanisms, and transferred patients. Pregnant patients presenting after penetrating trauma were compared to non-pregnant patients.

The primary outcome was mortality. Secondary outcomes included the incidence of GSWs and abdominal injuries. Demographic data included age, comorbidities, vitals upon arrival, and injury profile including injury severity score (ISS). Comorbidities included hypertension, congestive heart failure, chronic obstructive pulmonary disease, cirrhosis, diabetes, chronic kidney disease, smoking, psychiatric disorder, alcohol use disorder, and substance use disorder. Vitals on arrival were categorized as binary variables by the following: hypotension (systolic blood pressure < 100 mmHg), tachypnea (respiratory rate > 22 breaths/min), and tachycardia (heart rate > 120 bpm). These parameters were defined based on review of the literature on obstetric emergencies. Pregnant patients have a higher blood volume and so the standard threshold for hypotension (SBP < 90 mmHg) is not applicable to pregnant patients [[5], [6], [7], [8]]. The analyzed types of injury were stratified by anatomical location, including thoracic, gastrointestinal, genitourinary tract, neurological, and orthopedic injury. Severe abdominal injury was defined by an abbreviated injury scale (AIS) grade 3 or higher.

Clinical outcomes measured included emergent operations, and ventilator days. In-hospital complications such as cardiac arrest, catheter-associated urinary tract infection (CAUTI), central line-associated bloodstream infection (CLABSI), deep vein thrombosis, pulmonary embolism, unplanned intubation, acute respiratory distress syndrome (ARDS), ventilator-associated pneumonia, sepsis, cerebrovascular accident, post-operative wound infection, and unplanned return to the operating room (OR) were also recorded.

Frequency statistics were performed for all variables of interest, and a chi-square test and Man-Whitney-*U* test were used to compare categorical and continuous variables, respectively. Categorical data were reported as percentages, and continuous data were reported as medians with interquartile range (IQR) or as means with standard deviation.

A multivariable logistic regression model for mortality was performed, adjusting for age, hypotension on arrival, and ISS. These variables were chosen from collaborative discussions among the coauthors and review of the literature [[2], [3], [4]]. The adjusted risk of mortality was reported with an odds ratio (OR) and 95 % confidence intervals (CI). All *p*-values reported were two-sided with a statistical significance level of <0.05. All analyses were performed with IBM SPSS Statistics for Windows (Version 29, IBM Corp., Armonk, NY).

## Results

Out of 25,812 female patients <50 years of age sustaining penetrating trauma, 531 (2.1 %) were PTPs. The majority of PTPs were Black, and there was a greater proportion of Black PTPs than non-PTPs (56.1 % vs. 40.5 %, *p* < 0.001). PTPs were younger (median 26 vs 30 years-old, p < 0.001) and had decreased rates of pre-existing hypertension (3.2 % vs. 6.8 %, *p* = 0.001), diabetes (1.3 % vs. 3.2 %, *p* = 0.02), smoking (20.1 % vs. 29.2 %, p < 0.001), psychiatric disorder (11.2 % vs. 18.1 %, p < 0.001), and alcohol use disorder (2.1 % vs. 4.3 %, *p* = 0.01) compared to non-pregnant patients (Table 1). The overall rate of GSWs for PTPs was 7.6 % compared to 5.8 % in non-pregnant patients (p < 0.001). Vitals on admission for PTPs and non-pregnant patients were similar, except for a higher rate of tachypnea in PTPs (30.7 % vs. 25.7 %, p = 0.01) (Table 2).

**Table 1:** Demographics and comorbidities of non-pregnant versus pregnant penetrating trauma patients.

Characteristic	Non-pregnant	Pregnant	*P* value
	(*n* = 25,281)	(*n* = 531)	
Age, year, median (IQR)	30 (24–38)	26 (22–30)	**<0.001**
Race, n (%)			
Black	10,230 (40.5 %)	298 (56.1 %)	**<0.001**
White	11,679 (46.2 %)	168 (31.6 %)	**<0.001**
Asian	303 (1.2 %)	8 (1.5 %)	0.52
Comorbidities, n (%)			
Hypertension	1706 (6.8 %)	17 (3.2 %)	**0.001**
Congestive heart failure	56 (0.2 %)	1 (0.2 %)	0.88
COPD	290 (1.2 %)	3 (0.6 %)	0.21
Cirrhosis	28 (0.1 %)	0 (0.0 %)	0.44
Diabetes	806 (3.2 %)	7 (1.3 %)	**0.02**
Chronic kidney disease	24 (0.1 %)	0 (0.0 %)	0.50
Smoking	7350 (29.2 %)	106 (20.1 %)	**<0.001**
Psychiatric disorder	4570 (18.1 %)	59 (11.2 %)	**<0.001**
Alcohol use disorder	1081 (4.3 %)	11 (2.1 %)	**0.01**
Substance use disorder	3409 (13.5 %)	63 (12.0 %)	0.29

IQR = interquartile range, ISS = injury severity scale, ADHD = attention deficit hyperactive disorder, COPD = chronic obstructive pulmonary disease.

Significant P values (P < 0.05) are bolded.

**Table 2:** Injury profile of non-pregnant versus pregnant penetrating trauma patients.

Characteristic	Non-pregnant	Pregnant	P value
	(n = 25,281)	(n = 531)	
Mechanism of injury, n (%)			
Gunshot wound	11,636 (46.0 %)	341 (64.2 %)	**<0.001**
Stab wound	10,635 (42.1 %)	157 (29.6 %)	**<0.001**
Vitals, n (%)			
Hypotension (SBP <100 mmHg)	2891 (11.4 %)	68 (12.8 %)	0.33
Tachypnea (RR >22 breaths/min)	6243 (25.7 %)	157 (30.7 %)	**0.01**
Tachycardia (HR >120 bpm)	4048 (16.4 %)	83 (16.1 %)	0.85
ISS, median (IQR)	4 (1–10)	5 (1–10)	**0.02**
Thoracic injury, n (%)			
Heart	255 (1.0 %)	7 (1.3 %)	0.48
Lung	3021 (11.9 %)	62 (11.7 %)	0.85
Spleen	344 (1.4 %)	11 (2.1 %)	0.16
Gastrointestinal injury, n (%)	2149 (8.5 %)	33 (6.3 %)	**0.04**
Severe abdominal injury^a^	1924 (7.6 %)	52 (9.8 %)	0.06
Liver	1183 (4.7 %)	20 (3.8 %)	0.32
Stomach	379 (1.5 %)	9 (1.7 %)	0.71
Small intestine	932 (3.7 %)	27 (5.1 %)	0.09
Colon	885 (3.5 %)	31 (5.8 %)	**0.004**
Rectum	131 (0.5 %)	2 (0.4 %)	0.65
Genitourinary tract injury, n (%)			
Kidney	444 (1.8 %)	11 (2.1 %)	0.59
Bladder	143 (0.6 %)	1 (0.2 %)	0.25
Neurological injury, n (%)			
Brain	1243 (4.9 %)	27 (5.1 %)	0.86
Spinal cord	308 (1.2 %)	12 (2.3 %)	**0.03**
Orthopedic injury, n (%)			
Pelvic fracture	551 (2.2 %)	12 (2.3 %)	0.90
Upper extremity fracture	2389 (9.4 %)	68 (12.8 %)	**0.009**
Lower extremity fracture	1942 (7.7 %)	47 (8.9 %)	0.32

SBP = systolic blood pressure, RR = respiratory rate, HR = heart rate, bpm = beats per minute, AIS = abbreviated injury scale.

Significant P values (P < 0.05) are bolded.

a Defined as AIS grade 3 or above.

### Injury patterns in PTPs

PTPs had a higher median ISS (5 [IQR 1–10] vs. 4 [IQR 1–10], *p* = 0.02) and rates of overall gastrointestinal injury (8.5 % vs. 6.3 %, *p* = 0.04) and colon injury (5.8 % vs. 3.5 %, *p* = 0.004). However, PTPs had a statistically similar rate of severe abdominal injuries compared to non-pregnant patients (9.8 % vs. 7.6 %, *p* = 0.06). Notably, PTPs had higher rates of spinal cord injury (2.3 % vs. 1.2 %, *p* = 0.03) as well as upper extremity fractures (12.8 % vs. 9.4 %, *p* = 0.009) (Table 2).

### Outcomes of penetrating trauma in PTPs

Compared to non-PTPs, PTPs had a statistically similar in-hospital mortality rate (7.5 % vs. 5.6 %, *p* = 0.052). However, PTPs had an increased rate of in-hospital cardiac arrest (2.1 % vs. 1.0 %, *p* = 0.02) compared to their non-pregnant counterparts. Otherwise, the overall complication rate during hospitalization was similar between the two cohorts (4.7 % vs. 3.8 %, *p* = 0.28) (Table 3).

**Table 3:** Outcomes for non-pregnant versus pregnant penetrating trauma patients.

Outcomes	Non-pregnant	Pregnant	P value
	(n = 25,281)	(n = 531)	
pRBC transfusion, n (%)	3063 (12.1 %)	66 (12.4 %)	0.83
Plasma transfusion, n (%)	1790 (7.1 %)	39 (7.3 %)	0.81
Complications, n (%)	959 (3.8 %)	25 (4.7 %)	0.28
Cardiac arrest	253 (1.0 %)	11 (2.1 %)	**0.02**
CAUTI	29 (0.1 %)	1 (0.2 %)	0.62
CLABSI	4 (0.0 %)	0 (0.0 %)	0.77
Deep vein thrombosis	120 (0.5 %)	2 (0.4 %)	0.74
Pulmonary embolism	46 (0.2 %)	0 (0.0 %)	0.33
Unplanned Intubation	81 (0.3 %)	2 (0.4 %)	0.82
ARDS	28 (0.1 %)	1 (0.2 %)	0.60
Ventilator-associated pneumonia	39 (0.2 %)	1 (0.2 %)	0.84
Sepsis	33 (0.1 %)	0 (0.0 %)	0.41
Cerebrovascular accident	30 (0.1 %)	0 (0.0 %)	0.43
Unplanned return to OR	224 (0.9 %)	7 (1.3 %)	0.30
Post-operative wound infection, n (%)			
Superficial SSI	43 (0.2 %)	2 (0.4 %)	0.26
Deep SSI	42 (0.2 %)	0 (0.0 %)	0.35
Organ space SSI	54 (0.2 %)	0 (0.0 %)	0.29
Emergent operation, n (%)	7042 (27.9 %)	146 (27.5 %)	0.86
Ventilator days, median (IQR)	2 (1–4)	2 (1–4)	0.62
Mortality, n (%)	1408 (5.6 %)	40 (7.5 %)	0.052

pRBC = packed red blood cells, CAUTI = catheter-associated urinary tract infection, CLABSI = central line-associated bloodstream infection, ARDS = acute respiratory distress syndrome, OR = operating room, SSI = surgical site infection, LOS = length of stay, ICU = intensive care unit, IQR = interquartile range.

Significant P values (P < 0.05) are bolded.

### Multivariable analysis

On multivariable analysis, PTPs had similar associated risk of death (OR = 1.08, CI 0.65–1.77, *p* = 0.78) compared to their non-pregnant counterparts.

## Discussion

Unfortunately, penetrating trauma remains a major concern for PTPs in the United States. To our knowledge, this study is the first to utilize national data to analyze the injury patterns and clinical outcomes of PTPs presenting after penetrating trauma. We found a higher rate of gastrointestinal injuries in PTPs compared to non-pregnant female patients, challenging the proposed cushioning effect of the gravid uterus referenced in previous studies [6,7]. In addition, PTPs presented commonly with GSWs. Despite these disparities, this study revealed no significant differences in the associated risks of mortality or complications when comparing pregnant to non-pregnant trauma patients.

Pregnancy may impact injury patterns after penetrating trauma. The theory that the enlarged, muscular uterus acts as a protective shield against injury to visceral organs is contested by our findings, particularly given the observed increase in colon injuries among PTPs [6,9]. This challenges any notion of the uterus providing a “cushion effect.” Shah et al. purported that while the superiorly displaced visceral organs in pregnancy are less likely to be injured overall, they may be at greater risk when penetrating trauma involves the upper abdomen [9]. Interestingly, both PTPs and non-pregnant patients sustained similar rates of injury to other visceral organs and severe abdominal injuries. This similarity might be due to the nature of high-energy and high-velocity injuries, such as GSWs, which could override any potential protective benefits offered by the uterus. Essentially, the force and penetration capability of such injuries may be so significant that the protective cushioning effect of the uterus, if any, becomes negligible, resulting in comparable injury rates between pregnant and non-pregnant patients. It is unclear as to why the colon specifically is prone to injury after penetrating trauma in PTPs. This may be due to the anatomic changes that take place during pregnancy. As pregnancy progresses, the enlarging uterus exerts pressure on the abdominal cavity, leading to anterior displacement and potentially increased exposure of certain abdominal organs, such as the colon [7]. The cecum and transverse colon, being intraperitoneal and positioned relatively anteriorly in the abdominal cavity, are pushed further towards the abdominal wall. This displacement could make them more susceptible to injury from penetrating trauma, as they might be more directly in the path of penetrating trauma compared to other, more posteriorly located organs. The higher rate of colon injury in PTPs brings into question the effects of trauma on the fetus, as Santos et al. reported abdominal injury in PTPs as a risk factor for fetal delivery [10]. Thus, providers should be aware of this finding when evaluating penetrating trauma in the pregnant population.

Pregnancy may significantly impact the rate of non-abdominal injuries as well. The higher rate of upper extremity fractures in PTPs in our study is consistent with previous literature [11,12]. This could be secondary to the high rates of GSWs in PTPs, which are known to be associated with increased risk for severe bony injury [13]. We also identified a higher rate of spinal cord injury in PTPs, with case reports being some of the only studies looking into this type of injury [14]. This may also be related to anatomic changes during later stages of pregnancy. Increased stress on the spine due to lumbar lordosis and increased joint laxity, particularly as gestational age increases, could lead to more significant spine injuries when exposed to an equivalent level of traumatic force compared to non-pregnant patients [15].

PTPs may initially present in worse physiologic condition than non-PTPs. The increased rate of in-hospital cardiac arrests in PTPs could also be a consequence of high GSW rates as well as their lack of physiologic reserve. Increased blood volume in pregnancy can delay the signs of hemorrhage and shock, leading to more rapid decompensation [2,3]. Even though there were similar rates of PTPs and non-PTPs who were hypotensive and tachycardic on arrival, there is a high possibility that the PTPs in this cohort were more likely to decompensate than their non-pregnant counterparts. It is important for providers to be aware that due to the altered physiology in pregnancy, vital signs that are otherwise considered normal in non-PTPs may be concerning in PTPs.

PTPs may experience a disproportionally higher rate of certain types of traumatic mechanisms. Our study aligns with earlier reports, pointing to a distressingly high prevalence of GSWs among PTPs [9,16]. This correlates with significant social determinants of health, such as economic hardship and intimate partner violence (IPV), affecting pregnant patients. Luster et al. demonstrated that firearm violence in pregnancy predominantly impacts Black patients as well as patients already experiencing IPV [16]. Our study aligns with this as we found a higher rate of Black PTPs presenting after penetrating trauma, compared non-pregnant patients. Furthermore, the availability of loaded firearms in the home has been identified as a risk factor for pregnancy-related firearm violence [16,17]. The same population of pregnant patients who are more susceptible to penetrating trauma are also less likely to have adequate access to prenatal care and counseling [18]. This highlights the need for increased primary prevention efforts including screening and counseling for pregnant patients with risk factors for firearm injuries, including firearm ownership, firearm storage, and IPV.

Pregnancy has previously been thought to potentially impact mortality after trauma. Our study challenges the notion of pregnancy offering a protective “cushion effect” against mortality, a theory previously suggested in the literature [6,7]. The mortality rate and adjusted risk of death in PTPs and non-pregnant patients with penetrating trauma were found to be similar. Interestingly, our study noted a higher incidence of in-hospital cardiac arrest among PTPs, potentially attributable to the unique physiological changes during pregnancy that might obscure early signs of hemorrhage and shock, leading to quicker decompensation [19].

Despite its strengths in size and generalizability, this study has several limitations. As with other retrospective database studies, there is a possibility for coding errors and selection bias. Perhaps, the largest limitation of this study is the absence of pregnancy-related morbidities or injuries that are important to maternal-fetal outcomes such as incidences of uterine injury, placental abruption, fetal delivery, or fetal demise because the data were not available in the TQIP database [10,20]. Additionally, the lack of data on the gestational ages of PTPs prevented us from examining how injury patterns and outcomes might vary across different pregnancy stages. Finally, this study did not examine social determinants of health such as socioeconomic standing and IPV rates as the database is not granular enough.

## Conclusion

This national database analysis of PTPs demonstrated concerningly higher rates of firearm-related injury in PTPs compared to non-pregnant patients. The prevalence of firearm violence emphasizes the need for targeted primary prevention strategies for PTPs. Furthermore, the heightened vulnerability of PTPs to gastrointestinal injuries following penetrating trauma, potentially attributed to the gravid uterus's spatial changes, challenges any proposed “cushion effect.”

## CRediT authorship contribution statement

**Flora S. Park:** Writing – review & editing, Writing – original draft, Investigation, Formal analysis. **Jeffry Nahmias:** Writing – review & editing, Supervision, Investigation, Data curation, Conceptualization. **Jordan Shin:** Writing – review & editing, Formal analysis. **Ji Young Li:** Writing – review & editing, Formal analysis. **Cristobal Barrios:** Writing – review & editing, Supervision, Conceptualization. **Sebastian Schubl:** Writing – review & editing, Supervision, Conceptualization. **Areg Grigorian:** Writing – review & editing, Supervision, Methodology, Investigation, Formal analysis, Data curation, Conceptualization.

## Ethics approval

This study utilized a public deidentified national database, thus informed consent was not able to be obtained. Patient personal or identifying information has been excluded from the entirety of this manuscript.

## Funding

This research did not receive any specific grant from funding agencies in the public, commercial, or not-for-profit sectors.

## Declaration of competing interest

The authors report no conflicts of interest.

## Data Availability

Data may be made available upon request.

## References

[1] Lucas A.N., Tay-Lasso E., Zezoff D.C. (2024). Significant variation in computed tomography imaging of pregnant trauma patients: a retrospective multicenter study. Emerg Radiol.

[2] Oxford C.M., Ludmir J. (2009). Trauma in pregnancy. Clin Obstet Gynecol.

[3] Hill C.C., Pickinpaugh J. (2008). Trauma and surgical emergencies in the obstetric patient. Surg Clin North Am.

[4] Deshpande N.A., Kucirka L.M., Smith R.N. (2017). Pregnant trauma victims experience nearly 2-fold higher mortality compared to their nonpregnant counterparts. Am J Obstet Gynecol.

[5] Sakamoto J., Michels C., Eisfelder B. (2019). Trauma in pregnancy. Emerg Med Clin North Am.

[6] Wyant A.R., Collett D. (2013). Trauma in pregnancy: diagnosis and management of two patients in one. JAAPA.

[7] La Rosa M., Loaiza S., Zambrano M.A. (2020). Trauma in pregnancy. Clin Obstet Gynecol.

[8] Polena V., Huchon C., Varas Ramos C., Rouzier R., Dumont A., Fauconnier A. (2015). Non-invasive tools for the diagnosis of potentially life-threatening gynaecological emergencies: a systematic review. PLoS One.

[9] Shah A.J., Kilcline B.A. (2003). Trauma in pregnancy. Emerg Med Clin North Am.

[10] Santos J.W., Grigorian A., Lucas A.N. (2024). Predictors of fetal delivery in pregnant trauma patients: a multicenter study. J Trauma Acute Care Surg.

[11] El-Kady D., Gilbert W.M., Anderson J. (2004). Trauma during pregnancy: an analysis of maternal and fetal outcomes in a large population. Am J Obstet Gynecol.

[12] Schiff M.A., Holt V.L., Daling J.R. (2002). Maternal and infant outcomes after injury during pregnancy in Washington State from 1989 to 1997. J Trauma.

[13] Baum G.R., Baum J.T., Hayward D. (2022). Gunshot wounds: ballistics, pathology, and treatment recommendations, with a focus on retained bullets. Orthop Res Rev.

[14] Robertson K., Ashworth F. (2022). Spinal cord injury and pregnancy. Obstet Med.

[15] Han I.H. (2010). Pregnancy and spinal problems. Curr Opin Obstet Gynecol.

[16] Luster T., Loder R.T. (2023). Firearm injuries during pregnancy in the USA. Clin Pract.

[17] Burrell T.D., Voegtline K.M., Mistry K.B. (2021). An association between maternal intimate partner physical violence and a loaded firearm in the home. J Interpers Violence.

[18] Brown S.S. (1988). Prenatal care: Reaching mothers, reaching infants.

[19] Marco C.A., Sich M., Ganz E. (2022). Penetrating trauma: relationships to recreational drug and alcohol use. Am J Emerg Med.

[20] Aryan N., Grigorian A., Lucas A.N. (2023). Outcomes for advanced aged (35 and older) versus younger aged pregnant trauma patients: a multicenter study. Am J Surg.

